# Complications of Pancreatic Resection Reduced by Somatostatin

**DOI:** 10.1155/1993/64710

**Published:** 1993

**Authors:** C. W. Imrie

**Affiliations:** Royal Infirmary Glasgow G4 0SF Scotland


### COMPLICATIONS OF PANCREATIC RESECTION REDUCED BY SOMATOSTATIN

### ABSTRACT

*Buchler, M., Friess, H., Klempa, I., Hermanek, P., Sulkowski, U., Becker, H., Schafmayer, A., Baca, I., Lorenz, D., Meister, R., Kremer, B., Wagner, P., Witte, J., Zurmayer, E.L., Saeger, H-D., Rieck, B., Dollinger, P., Glaser, K., Teichmann, R., Konradt, J., Gaus, W., Dennler, H-J., Welzel, D. and Beger, H.G. (1992) Role of octreotide in the prevention of postoperative complications following pancreatic resection. The American Journal of Surgery; 163: 125–131.*

**Though morbidity and mortality rates following pancreatic resection have improved in recent years, they are still around 35% and 5%, respectively. Typical complications, such as pancreatic fistula, abscess, and subsequent sepsis, are chiefly associated with exocrine pancreatic secretion. In order to clarify whether the perioperative inhibition of exocrine pancreatic secretion prevents complications, we assessed the efficacy of octreotide, a long-acting somatostatin analogue.**

**We conducted a randomized, double-blind, placebo-controlled, multicenter trial in 246 patients undergoing major elective pancreatic surgery. Patients were stratified into a high-risk stratum (limited to patients with pancreatic and periampullary tumors) or low-risk stratum (patients with chronic pancreatitis). Patients received octreotide (3 × 100 μg) or placebo subcutaneously for 7 days perioperatively. Eleven complications were defined: death, leakage of anastomosis, pancreatic fistula, abscess, fluid collection, shock, sepsis, bleeding, pulmonary insufficiency, renal insufficiency, and postoperative pancreatitis.**

**Two hundred patients underwent pancreatic head resection, 31 patients underwent left resection, and 15 patients had other procedures. The overall mortality rate within 90 days was 4.5%, with 3.2% in the octreotide group and 5.8% in the placebo group. The complication rate was 32% in the patients receiving octreotide (40 of 125 patients) and 55% in patients receiving placebo (67 of 121 patients) ($p < 0.005$). In the patients in the high-risk stratum, complications were observed in 26 of the 68 (38%) patients treated with octreotide and in 46 of 71 (65%) patients given placebo ($p < 0.01$). Whereas in patients in the low-risk stratum, the complication rate was 25% (14 of 57 patients) in those treated with octreotide and 42% (21 of 50 patients) in patients given placebo ($p$ = NS).**

### PAPER DISCUSSION

KEY WORDS: Pancreatic resection, pancreatic carcinoma, somatostatin, octreotide

This is an excellent piece of work in which the high and low risk groups of patients have been well defined at the outset of the study. There is a slightly higher proportion of low risk patients having octreotide (50%) versus placebo (41%). However, this difference does not reach statistical significance. The multicentre German study (one Austrian centre) of the effect of 3 daily doses of subcutaneous octreotide (somatostatin analogue) given just before and for 7 days after major pancreatic surgery shows a distinct trend in favour of the patients who received the drug rather than placebo. The total number of 246 patients entering a randomised double blind placebo controlled multi-centre trial in less than one year is very creditable indeed. An average of 18 patients from each participating centre were recruited to the study and a median of 14 went forward to the stage of full active involvement in the trial. There is no similar piece of work in the literature to compare this with and it is encouraging that the high risk patients derived most benefit. Many authors have given octreotide as an adjunct in the management of pancreatic fistula and the minority in pancreatic pseudocyst management. The numbers in all these studies have been very small and in many cases almost anecdotal. In this particular piece of work the over all study had a mortality in the octreotide group of 3.2% (4 patients) and 5.8% in the placebo group. This difference was more marked in the high risk group of patients in which the relative mortalities were 2.9% and 9.9% respectively, so that there was greater than a threefold risk of death as a complication of surgery when octreotide was withheld. While it may be a minor point in such a large study it should be noted that two of the patients who were considered low risk and who received octreotide died while none of the patients in the low risk category within the placebo group died.

More information is specifically required about the 11 patients who died in the whole study, and in particular, it would be interesting to know why the two patients in the lower risk group receiving octreotide died. It may be valuable for the reader to know whether patients died at an early phase (within 10 days), intermediate (10–30 days) or later.

With regard to complications of the disease octreotide specifically reduced the expected complications of fistula, abscess and acute pancreatitis. Indeed regarding the analysis of the type and number of complications which is well outlined in Table 3, the only one of all the complications which was not reduced in the octreotide group of patients was bleeding.

On the subject of complications, 40 of the 246 patients had pancreatic duct occlusion with Ethibloc and they had a higher rate of fistula formation, but there is no breakdown anywhere in the paper to determine whether octreotide seemed to be beneficial or not for that subgroup.

One omission from the study is the description of what were considered standard prophylactic measures against the development of post-operative pulmonary embolism. Was there a standard protocol for this? This may be of some importance, especially when one considers that blood loss appeared to be a greater problem in the octreotide group. If heparin prophylaxis was standard, then the sporadic interaction between somatostatin and heparin may be a matter warranting consideration.

Overall the study is very impressive and the conclusion that octreotide be given to all patients undergoing high risk pancreatic surgery is a reasonable one.

Mr. C.W. Imrie
Consultant Surgeon
Royal Infirmary
Glasgow G4 0SF
Scotland

### SURGERY AND INTERVENTIONAL RADIOLOGY FOR BENIGN BILE DUCT STRICTURES

### ABSTRACT

*Schweizer, W.P., Matthews, J.B., Baer, H.U., Nudelmann, L.I., Triller, J., Halter, F., Gertsch, P. and Blumgart, L.H. (1991) Combined surgical and interventional radiological approach for complex benign biliary tract obstruction. British Journal of Surgery; 78: 559–563.*

**In patients with complicated high benign biliary strictures surgical technique alone cannot exclude the possibility of recurrent problems, and hepatic atrophy/ hypertrophy, portal hypertension and intrahepatic stones may all complicate surgical management. A multidisciplinary approach to these complex cases, which minimizes the need for repeated surgical interventions, has been pursued. Roux-en-Y hepaticojejunostomy was performed and an extended limb of the jejunum brought to the abdominal wall to allow access for later radiological intervention. Over a 30-month period 58 biliary-enteric anastomoses for benign disease were performed. Seventeen of these 58 patients were managed using the combined approach. Ten of these 17 patients had complex postcholecystectomy strictures and seven had strictures resulting from inflammatory disease, hepatic resection or congenital problems. A new classification of results of management of bile duct strictures is proposed. Seven patients were classified as 'excellent', six 'good', two 'fair' and two 'poor'. Results were obtained at a mean follow-up of 16 months and it seems likely that in some patients major surgical reinterventions were avoided.**

### PAPER DISCUSSION

KEY WORDS: Bile duct stricture, interventional radiology, access loop

Standard surgical techniques of biliary reconstruction offer a high probability of cure for the majority of patients with benign extrahepatic biliary strictures[1–4]. The technical difficulties encountered with high or recurrent strictures are simplified by using a left hepatic duct approach as first described by Hepp and Couinand[5]. However, when a Roux-en-Y jejunal loop is used for the biliary-enteric anastomosis, endoscopic access to the intrahepatic biliary tree is frequently lost. Access is essential in patients with complex recurrent intrahepatic strictures or stones where further problems are anticipated[6]. In the paper under review, Schweizer *et al.* have used percutaneous radiological techniques in selected patients with complex biliary problems as part of a combined approach with surgery using a Roux-en-Y hepaticojejunostomy and creation of an access loop for long-term dilatation. This dual approach is particularly suitable in patients who have had multiple previous operative attempts at repair or portal hypertension, segmental or lobar atrophy and hypertrophy, biliary infection and the presence of intrahepatic strictures and/or calculi which increase the difficulty and risk of surgery[6]. The authors leave a percutaneous silicone tube within the afferent jejunal loop for post-operative

